# Putting health status guided COPD management to the test: protocol of the MARCH study

**DOI:** 10.1186/1471-2466-13-41

**Published:** 2013-07-04

**Authors:** Janwillem Kocks, Corina de Jong, Marjolein Y Berger, Huib AM Kerstjens, Thys van der Molen

**Affiliations:** 1 Department of General Practice, University of Groningen, University Medical Center Groningen, Antonius Deusinglaan 1, 97136 AV, Groningen, the Netherlands; 2 Department of Pulmonary Diseases, University of Groningen, University Medical Center Groningen, Antonius Deusinglaan 1, 97136 AV, Groningen, the Netherlands; 3 Groningen Research Institute for Asthma and COPD (GRIAC), University Medical Center Groningen, Groningen, The Netherlands

## Abstract

**Background:**

Chronic Obstructive Pulmonary Disease (COPD) is a disease state characterized by airflow limitation that is not fully reversible and usually progressive. Current guidelines, among which the Dutch, have so far based their management strategy mainly on lung function impairment as measured by FEV_1_, while it is well known that FEV_1_ has a poor correlation with almost all features of COPD that matter to patients. Based on this discrepancy the GOLD 2011 update included symptoms and impact in their treatment algorithm proposal. Health status measures capture both symptoms and impact and could therefore be used as a standardized way to capture the information a doctor could otherwise only collect by careful history taking and recording. We hypothesize that a treatment algorithm that is based on a simple validated 10 item health status questionnaire, the Clinical COPD Questionnaire (CCQ), improves health status (as measured by SGRQ) and classical COPD outcomes like exacerbation frequency, patient satisfaction and health care utilization compared to usual care based on guidelines.

**Methods/Design:**

This hypothesis will be tested in a randomized controlled trial (RCT) following 330 patients for two years. During this period general practitioners will receive treatment advices every four months that are based on the patient’s health status (in half of the patients, intervention group) or on lung function (the remaining half of the patients, usual care group).

**Discussion:**

During the design process, the selection of outcomes and the development of the treatment algorithm were challenging. This is discussed in detail in the manuscript to facilitate researchers in designing future studies in this changing field of implementation research.

**Trial registration:**

Netherlands Trial Register, NTR2643

## Background

Chronic Obstructive Pulmonary Disease (COPD), a common preventable and treatable disease, is characterized by persistent airflow limitation that is usually progressive and associated with an enhanced chronic inflammatory response of the airways and the lungs to noxious particles or gases [1]. COPD has a considerable impact on health status [2]. Most guidelines, amongst which the 2003 Global initiative for Chronic Obstructive lung Diseases (GOLD) guidelines [3], and the Dutch GP guideline [4], have based severity categorization on lung function impairment, more specifically the FEV_1_. It is, however, well known that the FEV_1_ has a poor correlation with almost all patient reported outcomes in COPD and therefore the impact the disease has on the patient [5].

The GOLD strategy document update December 2011 [6] is the first update in which symptoms and exacerbations are included in patient assessment and severity grading. However a detailed management strategy is not included. As far as the authors know, there currently is no study in which the severity grading is tested via prospective algorithms. This may be one of the reasons that the GOLD update has not yet been incorporated in national guidelines.

Health status instruments have been developed specifically to assess disease severity, measure disease impact and to evaluate treatment. The use of validated health status instruments in daily clinical practice offers a wide range of opportunities. Information can be collected in a standardized manner prior to consultation. This may help decrease the known underestimation by clinicians of the impact of the disease and its treatment on the patients quality of life [7,8] and make it easier to review the patients condition over longer periods of time [9]. Studies have also shown that patient satisfaction is improved and patient opinions are more positive when quality of life questionnaires form part of routine practice [8,10]. High patient satisfaction is known to lead to superior compliance [11,12], to more promptly seeking medical care [13] and to retaining a higher amount of information [14]. One of the important prerequisites for using a questionnaire in routine clinical care, is its validation on individual patient level. Most questionnaires have solely been validated for the use in groups of patients. Validation on the individual level requires a different methodology. In the field of COPD this is currently only performed for the CCQ [15,16].

The 2003 GOLD guidelines, on which Dutch national guidelines are based, advocate a stepwise algorithm based on FEV_1_ level, differentiation is only recommended on the level of pharmacological treatment recommendations. All non-pharmacological recommendations are identical for all levels of severity, limiting individual differentiation. We propose that health status instruments provide the opportunity for individually tailored advices, focusing on functional status, mental status and symptoms. A form of tailoring that is, in a certain way, akin to the rising interest in pheno typing patients to target interventions more effectively [17].

Studies carried out in routine clinical practice show promising results regarding the feasibility of the use of health status instruments and their influence on the consultation, however until now these studies have not been able to show consistent benefits on outcomes for patients with COPD [18-20]. These ambiguous results might be due to differences in questionnaires used and in the way studies were performed. Studies that test the clinical effectiveness of health status instruments have used a large variety of tools, settings, and outcome parameters [18-26]. However none of these studies used a clear algorithm on how to interpret the outcome of health status measures nor did they feature clear advice regarding patient management.

The 2013 GOLD update includes both the COPD Assessment test (CAT) and the Clinical COPD Questionnaire (CCQ) as health status measurements [1]. One of the advantages of the CCQ over the CAT is its domains. This enables identification of the patients’ prime problem and thereby a focusing of the treatment on this problem. Also, the CCQ was rated to be more useful in primary care practice [27,28] and has been validated for use in individual patients [15].

We hypothesize that a treatment algorithm that is based on a simple validated measure of health status, the Clinical COPD Questionnaire (CCQ), improves health status (as measured on a separate scale) and secondary parameters like exacerbation frequency, patient satisfaction and health care utilization, when compared to usual care based on FEV_1_ level as per current GOLD guidelines.

The research questions addressed are:

1. Does a treatment algorithm that is based on CCQ measurements improve health status as measured by SGRQ over two years of use compared to usual care based on FEV_1_?

2. Does such a treatment algorithm improve other parameters of COPD care such as exacerbation frequency, mental health, health care utilization and direct medical costs compared to usual care based on FEV_1_?

This study combines the advantages of standardized health status measurement in routine clinical practice and of clear clinical treatment recommendations.

## Methods/Design

### Study design

The study will be a prospective randomized controlled trial with a follow-up duration of two year with two arms:

•intervention group with CCQ guided treatment proposals (CCQ group) and

•guideline group for whom treatment advice is based on FEV_1_ level according to Dutch National and GOLD guidelines (Usual Care, UC group).

**Figure 1:**
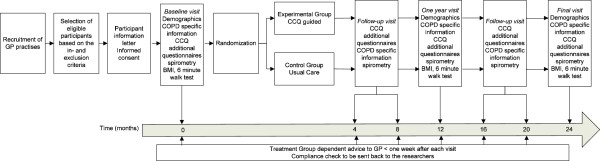
MARCH study flow chart.

The study flow-chart is represented in Figure 1. The study has been approved by the Medical Ethical Committee of the University Medical Center Groningen and is registered on the Dutch trial register (ISRCTN-register) with the identifier NTR2643.

### Duration

Patients will be followed up for 2 years and during that period there will be 7 visits, including a baseline and a final visit (Figure 1).

### Selection and recruitment

Local general practitioners will be contacted to participate in the study. When a general practitioner agrees to participate, he/she is asked to review his/her patient database for possible participants. The resulting eligible patients are sent a patient information leaflet and an informed consent form by their general practitioner. They will be asked by letter to return the informed consent form to their general practitioner if they wish to participate in the study (opt-in method). The patients will then be invited for the baseline visit. The inclusion criteria are a doctor’s diagnosis of COPD, age 40 years or above, a smoking history of at least 10 pack-years and a post bronchodilator FEV_1_/ forced vital capacity (FVC) <0.70. Exclusion criteria are a myocardial infarction less than 3 months ago, inability to read and understand the Dutch language, history of asthma or allergic rhinitis, regular use of oxygen, unstable or life-threatening co-morbid condition (as judged by the investigator) and dementia.

This study will take place in general practices in the Northern part of the Netherlands. All measurements including spirometry will be performed by a trained research nurse and will take place in or near the GP practices when possible.

### Randomization and blinding strategy

After inclusion into the study the patients will be randomized to the intervention or usual care group using a computerized randomization on individual patient level. The study will be performed in a double-blind fashion, patients and doctors will be blinded as well as the technicians that perform the measurements. A separate researcher will collect the data, and feed them in to a PC for a computerized treatment advice based on pre-defined criteria as per protocol. This advice will be sent to the doctor. Since the doctor will only see the resulting treatment advices, and not the measurement results they are based on, and since the treatment advices are compliant with the same national guide lines in both groups, albeit organized in a different fashion, blinding is maintained.

### Intervention

The actual intervention is the provision of treatment advices to the general practitioner based on health status in the CCQ group and based on FEV1 in the UC group.

### Algorithm development

#### Algorithm objective

CCQ group: the primary objective during the developmental phase was that the algorithm should result in a strategy that would treat the patient’s prime problem, reflected by the most impaired CCQ domain and not treat the remaining CCQ domains. At the next visit, it is assessed whether the impairments in the specific domain had improved sufficiently, the domain that is most impaired at that moment will guide the next period of treatment. The treatment intensity is guided by the CCQ total score, i.e. the overall impairment in health status.

UC group: this algorithm resulted directly from the treatment steps in the Dutch general practitioners guidelines.

#### Algorithm content

CCQ group: all current standard treatments options in the current Dutch general practitioners guidelines [4] were reviewed (JWHK) (pharmacological, stop-smoking, reactivation, counseling etc.) and rated on intensity of the treatment and expected possible effects of the treatment on each CCQ domain: symptoms, functional status or exercise capacity and mental state (JWHK, TvdM, HAMK). Subsequently the interventions were ordered according to intensity of the treatment and resources needed, e.g. for functional status this resulted in the following ordering: physical activity advices, out-patient reactivation, and rehabilitation.

UC group: all treatment steps in the Dutch general practitioners guidelines were directly translated into the algorithms.

#### Algorithm tuning

CCQ group: The concept treatment algorithm was discussed during an 45 minute meeting with pulmonologists and residents (n~15 present) working at the University Medical Center Groningen and during an one hour Groningen Research Institute for Asthma and COPD (GRIAC) research meeting. This GRIAC meetings are attended by both clinical and basic scientists of the departments of allergology, lab allergology and pulmonary diseases, epidemiology, general practice, molecular pharmacology, pathology, paediatric pulmonology and paediatric allergy, pulmonology and respiratory insufficiency. The concept algorithm was slightly altered as a result of these two meetings.

UC group: no further discussion nor fine tuning took place.

#### Algorithm feasibility

CCQ and UC group: to assess feasibility of the final algorithm, the algorithm was tested on databases of previous studies in the Wilhelmina Hospital Assen (n=38) and the Isala klinieken Zwolle (n=168).

#### Algorithm example

**Figure 2:**
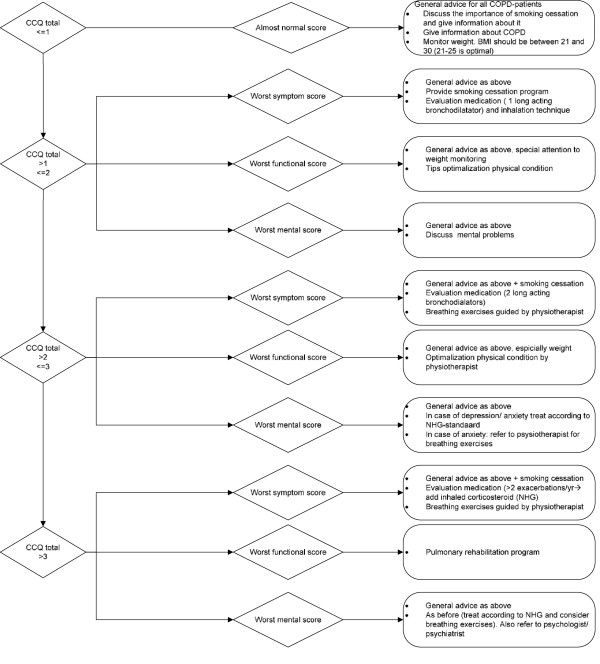
CCQ based treatment algorithm.

CCQ group: a high score on CCQ total score (>3, i.e. severely impaired) in combination with the highest score on the functional status domain leads to a pulmonary rehabilitation program advice, while a total CCQ score between 1 and 2 in combination with the highest score on the functional status domain leads to the provision of leaflets on healthy movement. A CCQ total score < 1 represents a very low burden of disease or good disease control, so no change in treatment advice is given. The final algorithm is displayed in Figure 2.

UC group: the advise is taken directly from the treatment steps in the Dutch general practitioners guidelines, a GOLD III score leads to the advise to use both a short acting and a long acting bronchodilator. In case of more than 2 exacerbations per year also the use of inhalation corticosteroids is advised.

### Measurements

#### Baseline visit and last visit

At each visit, the disease should be stable; in case of an exacerbation visits are postponed, there should be at least 6 six weeks between the end of the exacerbation and the visit. The following parameters are gathered at baseline and last visit:

•Patient demographics: age, gender, marital status, educational level, employment status.

•COPD specific information: smoking status, pack years, duration of COPD.

•Previous participation in a formal smoking cessation program, pulmonary rehabilitation or reactivation program.

•Co-morbidities, using the Charlson comorbidity index [29].

•Medication use and exacerbations in the last year. Exacerbations are defined as an increase in or new onset of more than one respiratory symptom (cough, sputum, sputum purulence, wheezing, dyspnea) with a duration of two or more days requiring treatment with an antibiotic and/or systemic steroid [30].

•Spirometry, pre- and postbronchodilator FEV_1_ in liters, FEV_1_ % predicted, FVC %predicted, and inspiratory capacity. The bronchodilator will be administered as salbutamol 4 times 100 microgram per metered dose inhaler with chamber device.

•Body Mass Index

•Functional exercise capacity as measured by the 6 minute walking test [31]. The patients are asked to walk up and down a level 30 meter walkway for 6 minutes. Breaks are allowed if necessary and recorded. Total distance walked is recorded as well as heart rate, blood pressure, Borg dyspnea score and oxygen saturation immediately before and after the test.

•Patient reported outcomes:

•The SGRQ is a 50-question, 76-item, health status scale for COPD patients. The SGRQ has 3 subscales: symptoms, activities and impact. The score ranges from 0 (best) to 100 (worst). The minimal clinically important difference is 4 points [32,33].

•The Clinical COPD Questionnaire is a 10-item health status scale measuring three domains: symptoms, functional status and mental state of COPD patients. Scores range from 0 (best) to 6 (worst). The minimal clinically important difference is 0.4 [34,35]. The CCQ has also been validated on the individual patient level [15].

•The modified Medical Research Council (mMRC) dyspnea scale [36]. This measures dyspnea on a scale of 0 (not breathless except when performing strenuous exercise) till 4 (too breathless to leave the house or breathless when dressing).

•The Hospital Anxiety and Depression Scale, a scale developed to identify anxiety disorders and depression among patients in non-psychiatric hospital clinics but also widely used outside the hospital. It is divided into an Anxiety subscale and a Depression subscale both containing seven items. Each question is answered on a 0 to three scale. A total score above 8 suggests the existence of pathology. A change of 1.5 in each domain score represents a clinically relevant change [37].

### During each follow-up visit

The following is collected during each follow-up visit: spirometry, pulmonary medication use, generic questionnaire about treatment offered and received, unscheduled visits to the GP or hospital because of pulmonary problems and patient reported outcomes: CCQ, SGRQ, mMRC, EuroQOL-5D and HADS.

### Advices to health care providers

After each visit the GP receives a treatment advice. Depending on the group to which the patient is randomized this is based either on the CCQ (CCQ group) or on the Dutch National guidelines (UC group). In order to check for compliance the GP is asked to report what treatment (pharmacological and non-pharmacological) was offered to the patient. If the GP deviates from this advice he or she is asked to the provide the reason for deviating.

### Outcomes

#### Primary outcomes

The primary outcome is change in SGRQ over time. Because the intervention is guided by the CCQ, a different health status instrument, the SGRQ, is used as primary outcome measure. Treatment of COPD patients in primary care is focused on improvement of health status and reduction of exacerbations. In this perspective it is a logical choice to use a health status questionnaire as an outcome measure.

#### Secondary outcomes

One of the secondary outcomes is the exacerbation frequency as indicated by medication use. This is one of the classical COPD outcomes, and exacerbations have a large impact on patients’ lives.

Other secondary outcome parameters are changes in CCQ score, 6 minute walking distance test, HADS, mMRC, lung function, and differences between the two groups in hospital admissions and mortality.

#### Economical outcome variables

Health care utilization and other direct medical costs are recorded. Data include medication use and all visits to the general practice, hospital, and other health care professionals involved in the management of COPD.

#### Sample size calculation

Sample size calculations are based on difference in change in health status between both groups. Because the intervention is guided by the CCQ, an alternative health status scale, the SGRQ, is used for the power-calculation. Based on 80% power to detect the minimal clinically important difference (4 points on the SGRQ) between the two groups, a sample size of 150 persons per group is needed. The standard deviation of the SGRQ total score in different samples is approximately10-17 (12 used in calculation) [38-42]. The alpha level was set at 0.05.

Taking dropouts into consideration, a sample size of 165 patients / group = 330 patients in total is aimed for.

### Statistical analysis

The primary outcome measures is the change in SGRQ over time. The SGRQ results in a total score and 3 subscale scores: symptoms, activities and impact. The SGRQ change in scores over the treatment period of the control group will be compared to that of the experimental group. The scores will be tested for normality. In case of normality the difference will be univariately tested with a student T-test and multivariately with a linear regression model. In case of deviation from normality the variable will be transformed to normality via a Box-Cox transformation and thereafter analyzed via student T-test and linear regression models.The multivariate models will be corrected for the following confounders: educational level, age, gender, current smoking, and FEV_1_. The number of exacerbations will be reported as weighted exacerbations rates (total number of exacerbations divided by the total person-time of follow up per group) [43-45]. Statistical significance of weighted rate ratios will calculated using a Poisson regression model. The secondary research outcomes will be tested in a similar fashion as the primary research question. The primary analyses will be based on the intention-to-treat principle. As secondary analyses, a per protocol analysis will be performed to increase insight in the data.

## Discussion

The objective of the MARCH study is to study whether a treatment algorithm that is based on health status as measured by CCQ improves health status as measured by SGRQ after two years of use compared to care based on FEV_1_ levels as per regular (GOLD) guidelines.

This study is based on the assumption that treatment based on problems that matter to patients (as reflected in a health status measurement) will have more positive effect on their life than treatment that is based on a single measurement that has little relation with their problems (FEV_1_).

The selection of an appropriate primary outcome measure for the current study was an important issue during the design process. The traditional primary outcome measure in COPD research is lung function, usually represented by the FEV_1_. The US Food and Drug Administration (FDA) and the European Medicines Association (EMA) still routinely require this in pharmaceutical trials. However, FEV_1_ has been found to have a very poor correlation with markers of COPD that seem to matter most to patients, such as exercise tolerance, symptoms and also health status [46,47]. Therefore, currently most researchers regard changes in patient centered outcomes such as health status, symptoms, exacerbations and functional status more important than changes in lung function [47]. Patient centered outcomes better reflect the complexity and the impact of the disease, and several aspects of health status predict clinically meaningful outcomes in COPD [48,49]. For instance, functional status as measured in health status questionnaires has been shown to predict exacerbations [50,51], hospital admissions [50-54] and mortality [55,56]. In most large scale COPD studies, health status is measured and demonstrated to improve after successful interventions, but it is seldom used as primary outcome. The situation is different in pulmonary rehabilitation studies where health status has been used as one of the primary endpoints [57].

Using health status as primary outcome measure in a study where the treatment in one arm is organized according to health status carries the risk of direct influence on the outcome. In order to reduce this potential methodological problem, a different health status questionnaire (SGRQ) is used in our study instead of the questionnaire that is used to guide the treatment (CCQ).

In the current study we decided to randomize on the patient level and not on the GP cluster level. This decision was made after careful evaluation of advantages and disadvantages of randomization on the individual and the cluster level. In this evaluation the following factors played a pivotal role. A large disadvantage of cluster randomization is the risk of selective inclusion, i.e. the physician is more likely to discover to which treatment group all his or her patients are allocated and this might, unconsciously, play a role in selecting patients for participation in the study. A second large disadvantage is the need for a much larger study population to maintain sufficient power. An additional power calculation assuming 10 COPD patients per practice, and a correlation of SGRQ within primary care practices scores of 0.14 (based on previous unpublished studies in our group), the total number of patients needed to achieve a power of 0.8 is 462. This constitutes an increase in patient number of 40%.

A disadvantage of randomizing at the individual level is the risk of contamination, loss of allocation concealment. This risk is present on both the patient level and on the physician level. On the patient level this is caused by the fact that several patients from one GP practice participate in this study and often patients in one practice know each other. Therefore patients in the control group might know patients that have been randomized into the intervention group and via that route receive information from the intervention group which they then might decide to use for themselves. However, we do not consider this to be a large risk in our study because the experimental treatment does not differ markedly from the usual care treatment, the same treatment elements are used albeit differently organized. In other words none of the patients will receive completely new and unexpected advices and therefore we expect them to conform to the recommendations given by their physicians.

The second level on which contamination might pose a risk for the study is the physician level, physicians might learn from the intervention and adjust their way of working. We try to circumvent this risk by supplying the physician with clear and individually tailored written practical advices. Physician and patients are routinely asked to report which treatment was given to each of the participants in the study giving us an accurate picture of whether or not contamination was present and if so the size of the problem.

Health care providers are not used to interpreting health status data. They need education and support to learn how to interpret the scores of health status instruments if they are to be successfully integrated into routine practice. Greenhalgh’s review of health status studies concluded that information should be fed back throughout the decision making process to all clinicians involved in the patient’s care and in a format they can make sense of and integrate in clinical decision making [23]. Health status scores should therefore be presented in a coherent clinically relevant format, with clear guidelines for interpretation and preferably with to-the-point recommendations. Based on Greenhalgh’s suggestions we incorporated in our study a clear treatment advice for the participating clinicians in order to avoid difficulties around the interpretations of health status scores.

Much effort was put in designing the treatment algorithms, because this is a pivotal part of the study design. During the design process choices without supporting evidence had to be made, this is because treatment based on health status is a novel concept and all previous studies were based on impairment of lung function as treatment criterion. By discussing the algorithm in different settings and with partners from various backgrounds we tried to reduce possible bias.

Vital for successful completion of the study is compliance of the care provider with the treatment advices. In the current Dutch GP practice the care for patients with chronic diseases is often transferred from the GP to the practice nurse. This applies also to implementing treatment advices. Practice nurses can achieve similar outcomes as doctors in chronic disease management [58]. Additionally, it has been demonstrated that practices in which the organization is optimal, guidelines are better adhered to [59]. Although this adds an extra layer in the process from measurement (lung function or health status) to effectuating the treatment, we are confident that in well organized practices with practice nurses, our advices will lead to similar results as with practices that do not work with practice nurses.

## Conclusions

This article describes the design of a double-blind randomized controlled trial in general practice that aims at demonstrating that COPD care can be improved by implementing a treatment algorithm based on a simple health care questionnaire. Considerations in choosing the primary end point, the randomization procedure and the design of the algorithm are described and result in decisions that both support the scientific robustness and feasibility of this study.

## Competing interests

The study was funded by an unrestricted grant by AstraZeneca.

JwhK: received grants from stichting Zorgdraad, and fees for lectures from GlaxoSmithKline. He received travel grants from GlaxoSmithKline, Chiesi, Boehringer Ingelheim. He acts as advisor for GlaxoSmithKline, Boehringer Ingelheim, Novartis.

CdJ: No competing interests.

MyB: no conflicting interests, she did not receive any financial support for her involvement in this study.

HamK: his institution has received fees per patient, consulting fees and travel support for from AstraZeneca, Boehringer Ingelheim, Pfizer, Takeda, Novartis, Almirall, and Chiesi.

TvdM: received grants from Chiesi, Astrazeneca, GlaxoSmithKline, MSD and fees for lectures from Astrazeneca, Allmirall, Glaxo Smith Kline, MSD, Nicomed. He acts as advisor for Astrazeneca, GlaxoSmith Kline, Mundifarma, MSD, Nicomed.

## Authors’ contributions

Jwhk, HamK and TvdM designed the study. Jwhk and CdJ drafted the manuscript. MyB, HamK and TvdM revised the manuscript critically for important intellectual content. All authors read and approved the final manuscript.

## Pre-publication history

The pre-publication history for this paper can be accessed here:

http://www.biomedcentral.com/1471-2466/13/41/prepub
